# Ultrasound-guided hydrostatic enema reduction for intussusception in children younger than 12 months: a systematic review and meta-analysis

**DOI:** 10.1007/s00383-026-06546-9

**Published:** 2026-07-31

**Authors:** Amani N. Alansari, Marwa Messaoud, Mohamed Sayed Zaazouee, Hanan Youssif, Ayman Albaghdady

**Affiliations:** 1 Department of Pediatric Surgery, Hamad Medical Corporation, P.O. Box 3050, Doha, Qatar; 2 Department of Pediatric Surgery, Fattouma Bourguiba University Hospital, Monastir, Tunisia; 3 Faculty of Medicine, Monastir University, Monastir, Tunisia; 4 Faculty of Medicine, Al-Azhar University, Assiut, Egypt; 5 Department of Pediatric Surgery, Benghazi Medical Center, Benghazi, Libya; 6 Department of Pediatric Surgery, Faculty of Medicine, Ain Shams University, Cairo, Egypt; 7 Faculty of Medicine, Badr University in Cairo, Cairo, Egypt

**Keywords:** Intussusception, Infants, Ultrasound-guided reduction, Hydrostatic enema, Nonoperative management

## Abstract

**Background and objectives:**

Intussusception is the most common cause of bowel obstruction in infancy and may lead to substantial morbidity. This systematic review and meta-analysis evaluated success, recurrence, and perforation rates of ultrasound-guided hydrostatic enema reduction (UGHR) in infants younger than 12 months.

**Methods:**

A systematic search was performed from database inception through January 2026. A random-effects model was applied to generate pooled estimates. Analyses, including subgroup analyses by sedation status, study design, and country income level, were conducted in R, and certainty of evidence was appraised.

**Results:**

Twenty-two studies, including 2656 infants, met the inclusion criteria. The pooled success rate was 80%, the recurrence rate was 8%, and the perforation rate was 2%. Subgroup analysis demonstrated no significant effect of sedation status or study design on outcomes, whereas income level was significantly associated with perforation risk. Substantial heterogeneity was observed across outcomes. Overall certainty of evidence was low.

**Conclusions:**

UGHR demonstrates a clinically meaningful success rate with an acceptable safety profile in infants under 12 months, supporting its role as a safe and effective non-surgical treatment in hemodynamically stable patients. Standardized global protocols and high-quality prospective multicenter studies are needed to optimize outcomes and strengthen the evidence base.

**Supplementary Information:**

The online version contains supplementary material available at 10.1007/s00383-026-06546-9.

## Introduction

Intussusception is the leading cause of intestinal obstruction in children aged 3 months to 6 years and one of the most common abdominal emergencies, with a global incidence of approximately 30–100 cases per 100,000 infants annually [1, 2]. It occurs when a proximal segment of bowel telescopes into a distal segment, potentially leading to serious complications such as venous congestion, ischemia, perforation, if not promptly diagnosed and treated [3]. The peak incidence occurs between 5 and 7 months of age, and approximately 60–75% of cases arise within the first year of life [2]. Ileocolic intussusception accounts for 80–90% of cases [4]. Although mortality has declined to below 1% in high-income countries due to early diagnosis and prompt intervention, case-fatality rates in low-resource settings still range from 8.5 to 14.3%, largely due to delayed presentation and limited access to timely care [5, 6].

Nonoperative reduction has become the cornerstone of treatment for hemodynamically stable children without signs of peritonitis or perforation [7]. Over the past several decades, image-guided enema reduction, using either pneumatic or hydrostatic techniques, has largely replaced primary surgical management in appropriate candidates [8]. Among these approaches, ultrasound-guided hydrostatic enema reduction (UGHR) has gained increasing attention because it avoids ionizing radiation, provides visualization of bowel reduction, and facilitates early recognition of complications [9, 10]. Particularly in infants younger than 12 months, in whom rapid clinical deterioration and bowel vulnerability are concerns, a safe and effective nonoperative approach is essential [11].

Despite its growing adoption, reported outcomes of UGHR vary widely, with success, perforation, and recurrence rates influenced by factors such as age, presenting features, symptom duration, patient selection, institutional experience, and regional practices [12–15], while most evidence comes from retrospective studies with limited randomized data, contributing to uncertainty in overall efficacy and safety estimates [16–19].

Infants younger than 12 months represent a clinically distinct subgroup. They often present with nonspecific symptoms such as irritability, vomiting, or lethargy, which may delay diagnosis [20]. In addition, anatomical and physiological differences in this age group may influence both reduction success and complication risk. These differences include a smaller intestinal diameter, thinner bowel wall, higher mobility of the cecum, and an immature immune response, all of which can affect both the presentation and the risk of perforation during reduction [21]. Previous reviews have frequently combined age groups or pooled pneumatic and hydrostatic techniques, limiting age-specific and modality-specific conclusions [10, 14].

Accordingly, this systematic review and meta-analysis was conducted to assess the effectiveness and safety of UGHR for pediatric intussusception in infants under 12 months of age. The primary outcomes were pooled success, perforation, and recurrence rates, while the secondary outcomes were subgroup analyses by sedation status, study design, and country income level.

## Methods

### Study design and reporting standards

This review and meta-analysis were undertaken following established methodological frameworks for evidence synthesis and were reported in accordance with the Preferred Reporting Items for Systematic Reviews and Meta-Analyses (PRISMA) statement and Cochrane handbook guidelines [22, 23]. The study was registered in PROSPERO (CRD420261294714).

### Search strategy

A systematic literature search was conducted across five databases: PubMed, Cochrane Library, Scopus, Web of Science, and Embase. The search approach integrated controlled vocabulary with relevant free-text terms pertaining to pediatric intussusception, infants, UGHR, and nonoperative management. All records from database inception to January 2026 were considered, without geographic restrictions. Additionally, the reference lists of included studies were manually reviewed to identify further eligible publications (Table S1).

### Eligibility criteria

Studies qualified for inclusion if they involved pediatric patients under 12 months of age with intussusception treated using UGHR. Eligible studies were required to report at least one of the following outcomes: success, perforation, or recurrence rates. Accepted study designs comprised observational studies (cohort, case–control, and cross-sectional), case series, and randomized controlled trials (RCTs). Only English-language publications were considered. Studies were excluded if they were review articles, case reports, conference abstracts or lacking full data.

### Study selection

All retrieved records were uploaded into EndNote 20, and duplicates were removed. Two reviewers independently screened titles and abstracts for relevance. Full texts of potentially eligible studies were obtained and evaluated according to predefined inclusion and exclusion criteria. Disagreements were settled through discussion and mutual agreement.

### Data extraction

Two reviewers independently collected data using a standardized form. Extracted variables included study characteristics (country and design), sample size, patient demographics, intussusception type, symptom duration, number of successful reductions, perforation events, recurrence events, and follow-up duration. Any inconsistencies between reviewers were resolved through discussion and consensus.

### Quality assessment

Methodological quality was assessed according to study design using validated appraisal tools. Observational cohort and case–control studies were evaluated using the Newcastle–Ottawa Scale (NOS) [24], which assesses selection methods, comparability, exposure/outcome ascertainment, and follow-up adequacy. The case series study was assessed using the National Institutes of Health (NIH) Quality Assessment Tool for Case Series Studies [25], evaluating clarity of objectives, population description, intervention reporting, outcome assessment, and statistical reporting. The cross-sectional study was appraised using the Newcastle–Ottawa Scale adapted for cross-sectional studies [26], focusing on representativeness, exposure and outcome assessment, confounding control, and statistical methods. The randomized controlled trial was evaluated using the Cochrane Risk of Bias tool, assessing domains including random sequence generation, allocation concealment, blinding, incomplete outcome data, and selective reporting [27]. Quality assessments were conducted independently by two reviewers, with disagreements resolved through consensus.

### Statistical analysis

A random-effects meta-analysis was used to address expected clinical and methodological heterogeneity. Pooled proportions with 95% confidence intervals were calculated for success, perforation, and recurrence rates. Heterogeneity was evaluated using the Cochran Q test and quantified with the* I*^2^ statistic, with values > 50% indicating substantial heterogeneity. Subgroup analyses were also performed based on sedation status, economic settings, and study design to investigate potential sources of heterogeneity. Publication bias was assessed visually through funnel plots and formally evaluated using Egger's regression test. All analyses were performed in R programming, and a *p*-value < 0.05 was considered statistically significant.

### Certainty of evidence

The certainty of evidence for each outcome was appraised using the Grading of Recommendations Assessment, Development and Evaluation (GRADE) framework [28], evaluating risk of bias, inconsistency, indirectness, and imprecision.

## Results

**Fig. 1:**
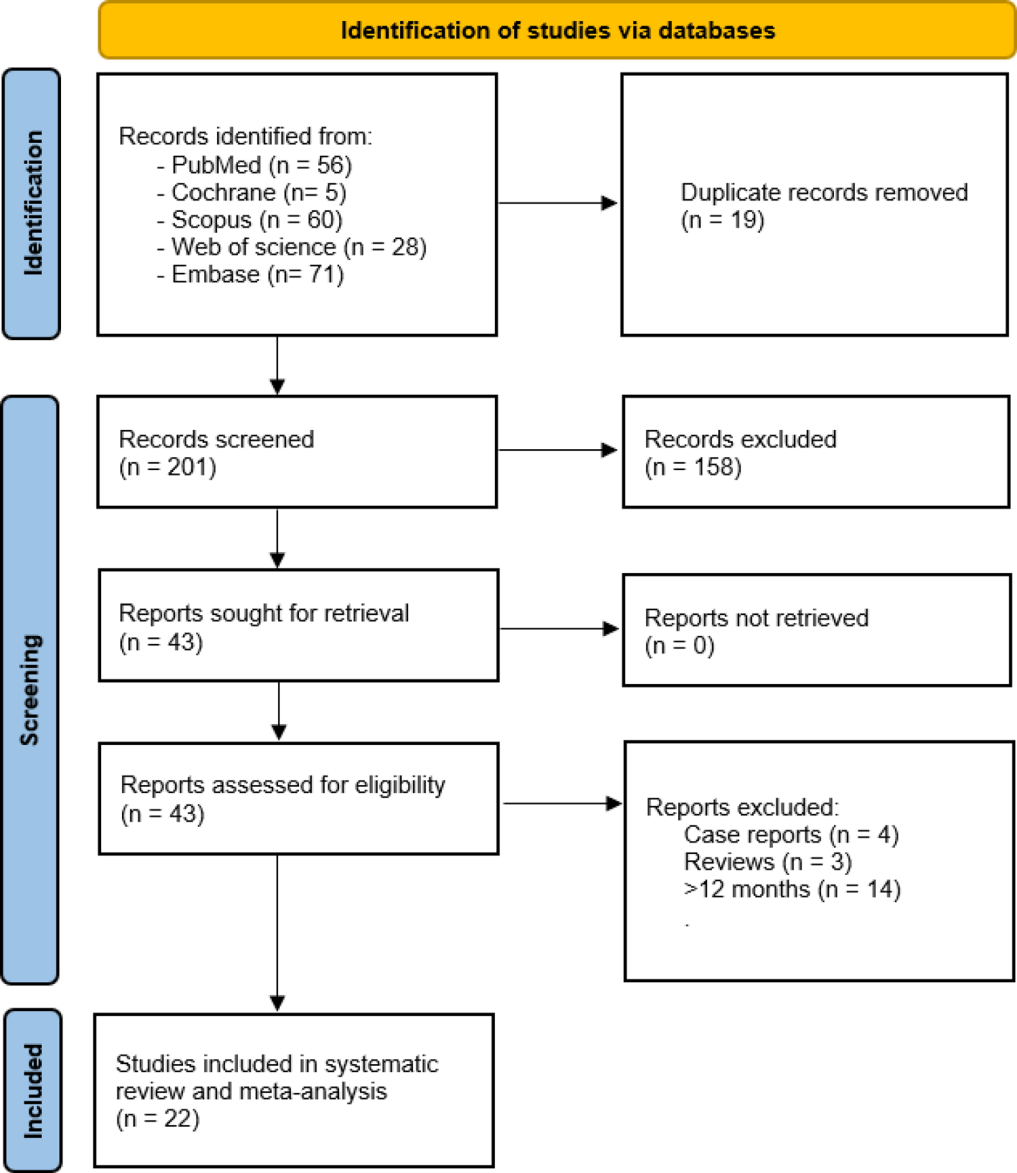
PRISMA flow diagram of the study selection process

A systematic search of five electronic databases, PubMed (n = 56), Cochrane (*n* = 5), Scopus (*n* = 60), Web of Science (*n* = 28), and Embase (*n* = 71), identified 220 records. After removing 19 duplicates, 201 records remained for title and abstract screening. Subsequently, 43 full-text articles were retrieved and evaluated for eligibility, all of which were successfully obtained. During the eligibility evaluation, studies were excluded due to being case reports (*n* = 4), review articles (n = 3), or not specific to our age restriction, exceeding 12 months (*n* = 14). Finally, 22 studies met the inclusion criteria [16–19, 29–46] (Fig. 1).

### Baseline and summary of the included studies

The included studies encompassed approximately a total of 2656 pediatric patients with sample sizes varying from 8 to 660 participants. Most investigations were retrospective cohort studies, alongside two prospective cohorts, one cross-sectional study, one prospective case series, one retrospective case–control study, and a single RCT. Studies spanned from 1994 to 2026 and represented diverse geographic regions, including China, India, Nigeria, Egypt, Nepal, Australia, Israel, Pakistan, Thailand, Ghana, Italy, and South Korea. Participants were infants younger than 12 months. Ileocolic intussusception was the most frequently reported subtype, often accounting for 80–100% of cases, whereas ileo-ileal and colo-colic variants were less common. Symptom duration varied considerably, commonly reported as < 24 h, 24–48 h, or > 48–72 h (Table 1).

**Table 1:** Summary of the included studies

ID	Reduction technique	Total Sample	Site	Study design	Study period	Age, months (mean)	Male, n	Intussusception site, n (%)	Duration of symptoms, hours
Qiang et al. (2026)	UGHR	257	China	Retrospective cohort study	January 2021–August 2025	< 12, 257(100%)	–	–	–
Soundharya et al. (2025)	UGHR	47	India	Retrospective cohort study	January 2016–December 2018	< 12, 47(45.63%)	–	Ileo-colic	< 48
Yadav et al. (2025)	UGHR	84	Nepal	Cross sectional study	February 2023–July 2024	< 12, 67(82.72%)	60	Ileo-colic, 74(88) Colo-colic, 8(9.5) Ileo-ileal, 2(2.4)	24, 30(37%) 24–48, 39(48%) > 48, 12(14.8%)
Tang et al. (2024)	UGHR	15	China	Retrospective cohort study	–	≤ 10.5, 1172 (23.6%)	–	–	–
Binu et al. (2023)	UGHR	108	Australia	Retrospective cohort study	2012–2020	12	–	Ileocolic, 108(100)	–
Chukwu et al. (2022)	UGHR	26	Nigeria	RCT	December 2018–August 2020	1 to 12	16	Ileo-colic, 26(100)	72 ± 48.9
	UGPR	26					19	Ileo-colic, 26(100)	76.8 ± 38.8
Ali et al. (2022)	UGHR	55	Pakistan	Retrospective case control study	–	12	34	–	< 72, 40(72.73%) > 72, 15(27.23%)
Zhang et al. (2022)	UGHR	71	China	Retrospective case control study	January 2019–April 2021	< 12, 71(28.4%)	–	Ilea-colic, 71(100)	≤ 24
Yu et al. (2021)	UGHR	178	China	Retrospective cohort study	–	< 12, 178(77%)	–	Ileo-colic, 178(100)	9 ± 5.33
Chukwubuike et al. (2020)	UGHR	20	Nigeria	Retrospective cohort study	October 2017–March 2019	< 12, 20(100%)	16	Ileocolic, 16(80) Ileo-ileal, 4(20)	< 24, 3(15%) 24–48, 8(40%) > 48, 9(45%)
Sarma et al. (2020)	UGHR	152	India	Retrospective cohort study	January 2017–December 2017	11	94 of 176	Ileocolic, 152(100)	Mean (48)
Sacks et al. 20200	UGHR	338	Israel	Retrospective cohort study	1998–2018	11.1	213	Ileocolic, 338(100)	–
Shen et al. (2018)	UGHR	660	China	Retrospective cohort study	January 2010–December 2017	< 12, 660(36.81%)	–	–	–
Talabi et al. (2018)	UGHR	35	Nigeria	Prospective cohort study	January 2016–June 2017	< 35, 35(77.78%)	–	Ileo-colic and colo-colic	mostly < 24
Eraki et al. (2017)	UGHR	50	Egypt	Retrospective cohort study	April 2011–April 2013	12	–	Ilea-colic and Ileo-ileal	< 24, 50(100%)
Khorana et al. (2015)	UGHR	59	Thailand	Retrospective cohort study	January 2006–December 2012	9	39	Ilea-colic and Ileo-ileal	–
	PR	111					75		–
He et al. (2014)	UGHR	288	China	Retrospective cohort study	Over 3 years	< 12, 169(58.68%)	168	mostly ascending colon	–
Atalabi et al. (2013)	UGHR	72	Egypt	Prospective cohort study	January 2005 – September 2013	< 12, 33(91.67%)	24	–	–
Renzo et al. (2012)	UGHR	9	Italy	Retrospective cohort study	2000–2009	< 12, 9(77%)	–	–	27.9 h ( range 6–72 h)
Mensah et al. (2011)	UGHR	18	Ghana	Retrospective cohort study	March 2008–December 2008	11.7	13	Ascending colon, 8(40) Transverse, 5(25) descending, 4(20)	–
Atalabi et al. (2007)	UGHR	8	Nigeria	Prospective case series study	July 2004–June 2006	7.75	6	–	Mean (32.64)
Choi et al. (1994)	UGHR	115	South Korea	Retrospective cohort study	April 1988–August 1992	10.9	82	Ileo-colic, 82(71.3)	–

*UGHR* Ultra-sound Guided Hydrostatic Reduction, *UGPR* Ultra-sound Guided Pneumatic Reduction, *PR* Pneumatic Reduction, *RCT* Randomized Controlled Trial, (–) Not Reported or Reported but Not Specified for Our Age Group

#### Primary analysis

##### Success rate

**Fig. 2:**
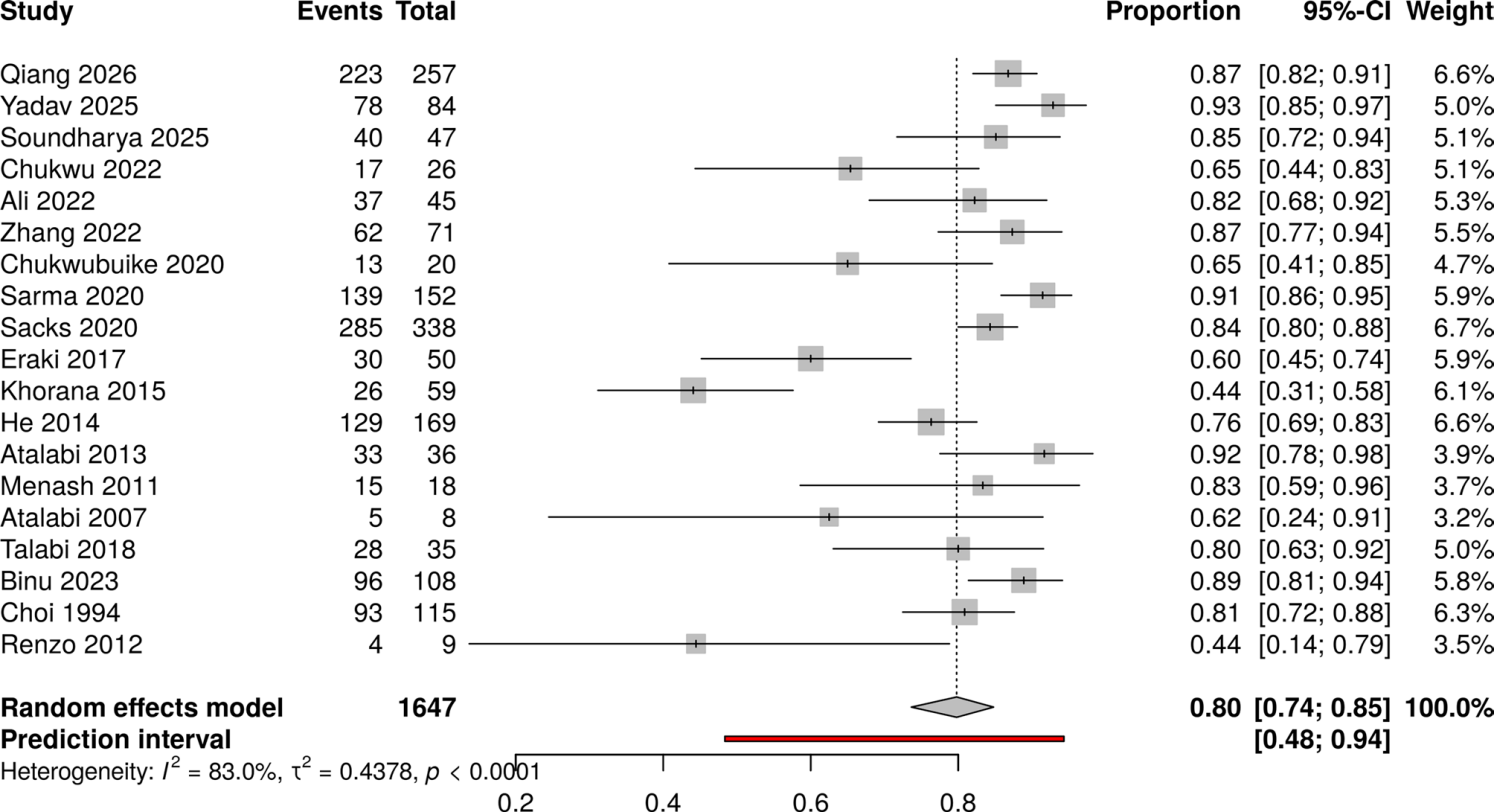
Forest plot of the pooled success rate

The pooled analysis of 19 studies demonstrated a high overall success rate of 80% (95% CI: 74–85%), based on 1353 successful reductions among 1647 patients. Individual study estimates ranged from 44 to 93%. Substantial heterogeneity was observed (*I*^2^ = 83%, *p* < 0.0001) (Fig. 2).

##### Recurrence rate

**Fig. 3:**
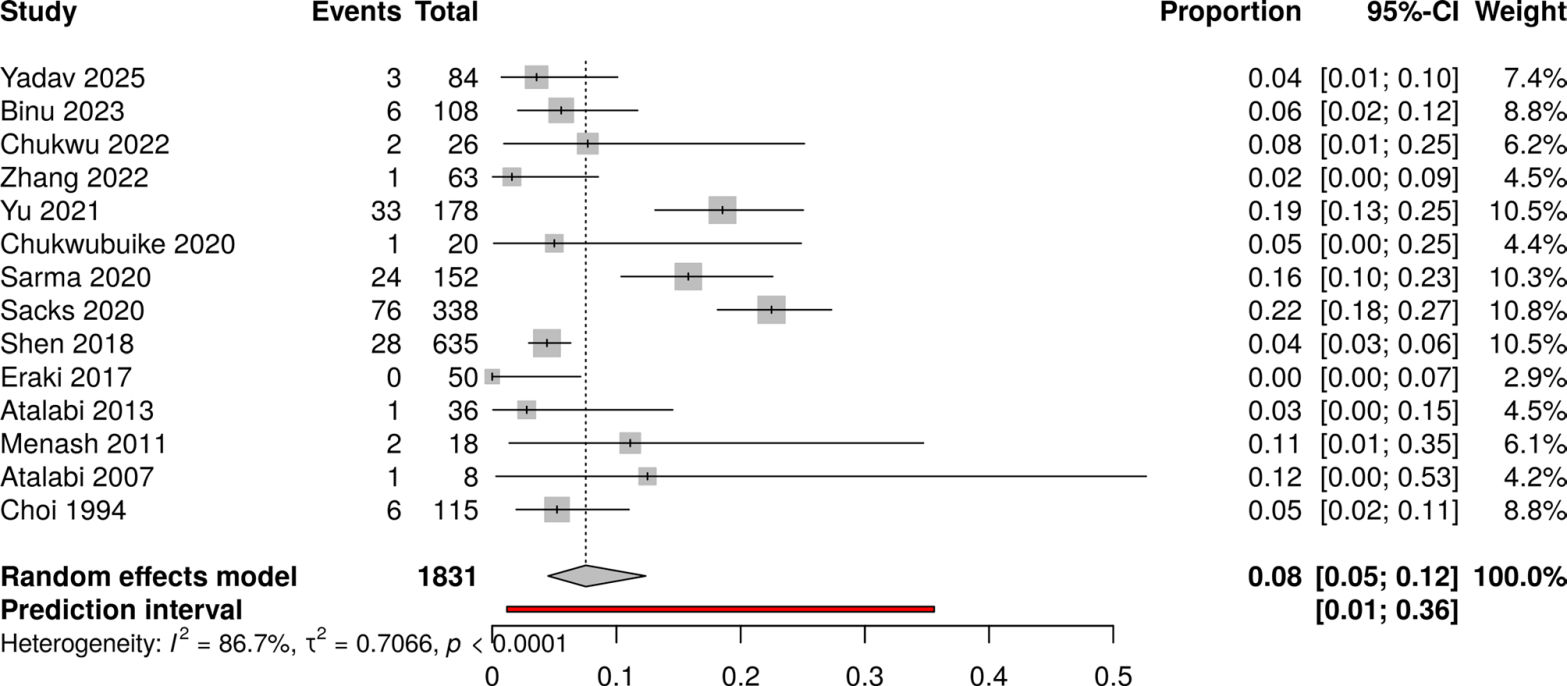
Forest plot of the pooled recurrence rate

Fourteen studies were included in the recurrence analysis, demonstrating an overall recurrence rate of 8% (95% CI: 5–12%), with 184 events among 1831 patients. Reported recurrence rates ranged from 0 to 22%, and heterogeneity was high (*I*^2^ = 86.7%, *p* < 0.001) (Fig. 3).

##### Perforation rate

**Fig. 4:**
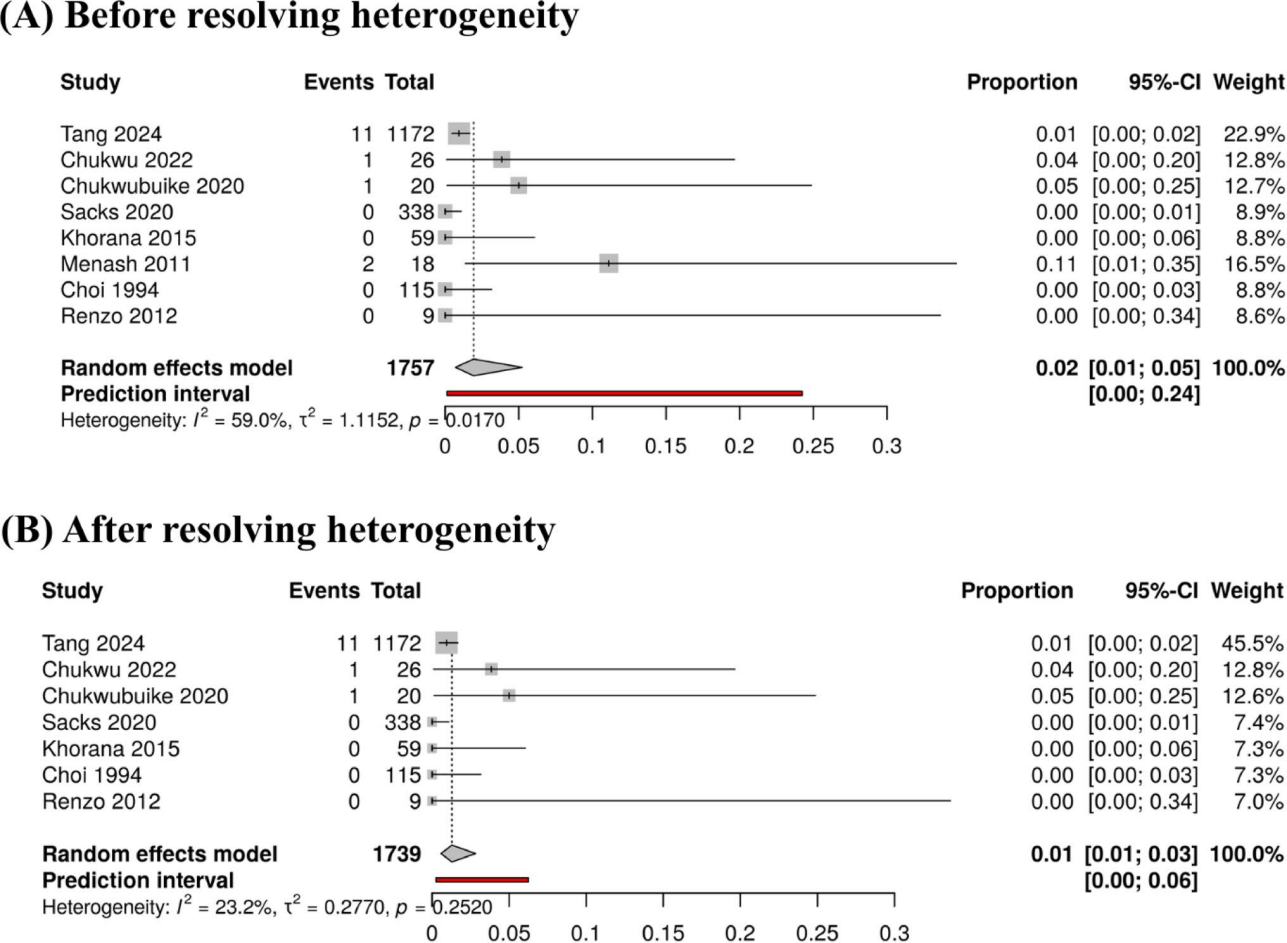
Forest plot of the pooled perforation rate

Eight studies contributed to the perforation analysis, with a pooled rate of 2% (95% CI: 1–5%), based on 15 events among 1757. Individual estimates ranged from 0 to 11%, with moderate to substantial heterogeneity (*I*^2^ = 59%, *p* = 0.017). After excluding the study by Mensah et al. (2011) to address heterogeneity, the pooled perforation rate decreased to 1% (95% CI: 1–3%), while heterogeneity decreased substantially (*I*^2^ = 23.2%) (Fig. 4).

#### Secondary analysis

##### Subgroup analysis of success rate

Sedation status, study design, and country income level were not associated with significant differences in success rate. Infants who received sedatives had a success rate of 73% (95% CI: 53–87; *I*^2^ = 92%) versus 82% (95% CI: 77–86; *I*^2^ = 71%) in those who did not (subgroup *p* = 0.2959). Likewise, success rates were comparable across study designs (*p* = 0.4125), including retrospective studies [80% (95% CI: 73–85; *I*^2^ = 85%)], single prospective cohort [80% (95% CI: 63–92)], and cross-sectional/case series studies [84% (95% CI: 41–97; *I*^2^ = 83.1%)], although the single RCT reported a lower rate of 65% (95% CI: 44–97). Similarly, no significant variation was observed by country income level (p = 0.8861), with pooled success rates of 81% (95% CI: 65–90; *I*^2^ = 79.5%) in high-income countries, 77% (95% CI: 63–87; I^2^ = 91.7%) in upper-middle-income countries, and 81% (95% CI: 72–88; *I*^2^ = 76.1%) in lower-middle-income countries (Figs. S4–S6).

##### Subgroup analysis of recurrence rate

Recurrence rates did not differ significantly by sedation status, study design, or country income level. Procedures performed without sedation showed a pooled recurrence rate of 5% (95% CI: 3–11; *I*^2^ = 74.2%) compared with 11% (95% CI: 5–24; *I*^2^ = 93.7%) in those using sedative agents (subgroup *p* = 0.1941). Similarly, recurrence remained comparable across study designs (p = 0.8025), including retrospective studies [8% (95% CI: 4–13; *I*^2^ = 89%)], cross-sectional/case series studies [5% (95% CI: 2–15; *I*^2^ = 18.3%)], and the single RCT [8% (95% CI: 1–25)]. No significant differences were also observed according to country income level (*p* = 0.7446), with recurrence rates of 12% (95% CI: 3–40; I^2^ = 92.4%) in high-income countries, 6% (95% CI: 2–16; *I*^2^ = 92.4%) in upper-middle-income countries, and 7% (95% CI: 4–14; I^2^ = 49.7%) in lower-middle-income countries (Figs. S7–S9).

##### Subgroup analysis of perforation rate

Perforation rates were not significantly affected by sedation status or study design, but differed significantly according to country income level. UGHR performed without sedation had a perforation rate of 1% (95% CI: 1–4;* I*^2^ = 28%), compared with 2% (95% CI: 0–12; *I*^2^ = 66.3%) in those performed with sedation (subgroup *p* = 0.7341). Likewise, no significant difference was observed by study design (*p* = 0.4854), with retrospective studies reporting a perforation rate of 2% (95% CI: 1–5; *I*^2^ = 62.5%) and the single randomized trial reporting 4% (95% CI: 0–20). In contrast, income level showed a significant subgroup effect (*p* = 0.0019), with the highest perforation rate in lower-middle-income countries [7% (95% CI: 3–17; *I*^2^ = 0%)], followed by upper-middle-income countries [1% (95% CI: 1–2;* I*^2^ = 0%)], and high-income countries 1% (95% CI: 0–22) (Figs. S10–S12).

### Quality assessment

Overall, the included studies demonstrated predominantly moderate to high methodological quality. Among the 19 observational cohort and case–control studies, six were classified as high quality, [17, 34, 37, 42, 44–46], demonstrating strong methodological rigor, clear case definitions, appropriate selection processes, and reliable outcome assessment. Ten studies were classified as moderate quality [16, 19, 30, 33, 35, 36, 38–41], most commonly limited by issues related to group comparability, potential confounding, or follow-up adequacy. Two studies were judged to be low quality due to weaknesses in study design and reporting [18, 31]. In addition, one case series was assessed as good quality, with clearly defined objectives and well-described results despite inherent design limitations [29]. The single cross-sectional study was rated as moderate quality [43]. Finally, one randomized controlled trial demonstrated overall moderate methodological quality, with some concerns related to blinding and potential sources of bias [16] (Tables S2–S5).

### Publication bias

Visual inspection of the funnel plots revealed slight asymmetry for the pooled success and recurrence rates. Egger's regression test, however, showed no significant evidence of funnel plot asymmetry for the success rate (intercept =  − 0.70, 95% CI: − 3.35 to 1.94; *p* = 0.609), indicating no statistically significant publication bias. In contrast, both the funnel plot and Egger's test suggested potential publication bias for the recurrence rate (intercept =  − 2.33, 95% CI: − 4.32 to − 0.34; *p* = 0.040). For the perforation rate, the funnel plot appeared largely symmetrical, and Egger's test likewise showed no evidence of funnel plot asymmetry (intercept = 0.66, 95% CI: − 1.29 to 2.61; *p* = 0.532), indicating no significant publication bias despite the observed moderate between-study heterogeneity (Figs. S10–S12).

### GRADE assessment

For success (19 studies), recurrence (14 studies), and perforation (8 studies), the overall certainty of evidence was rated as low. Although indirectness and imprecision were not considered serious, the evidence was downgraded due to concerns about risk of bias and substantial inconsistency driven by high heterogeneity (I^2^ > 70%) (Table S6).

## Discussion

### Summary of findings

This meta-analysis synthesized evidence from 22 studies encompassing approximately 2656 infants younger than 12 months who underwent UGHR for intussusception. Our analysis revealed a pooled success rate of 80%, a recurrence rate of 8%, and a perforation rate of 2%, all accompanied by substantial heterogeneity across contributing studies. Subgroup analyses showed that hydrostatic reduction success, recurrence, and perforation rates were generally consistent across sedation status and study design, with no significant differences between these categories. In contrast, country income level significantly influenced perforation risk, with higher rates observed in lower-middle-income settings, suggesting that resource-related factors may affect procedural safety.

The high overall success rate likely reflects the combined advantages of ultrasound visualization and the gentle, controlled pressure delivered through hydrostatic technique [9]. Unlike fluoroscopic or pneumatic approaches, ultrasound guidance allows the clinician to directly monitor bowel wall movement, detect residual intussusception, and identify early complications without exposing the infant to ionizing radiation, a particularly meaningful benefit in this age group [47, 48]. The relatively favorable success rate also resonates with the well-established principle that timely intervention correlates strongly with reduction success; many included studies enrolled infants with symptom durations under 24 h, a window known to be associated with higher reducibility [17, 39]. Conversely, the heterogeneity observed likely reflects genuine variability in clinical practice: differences in hydrostatic pressure limits, sedation protocols, operator experience, patient selection thresholds, and the proportion of cases with prolonged symptom duration all contribute to outcome variability across diverse international settings [12, 19]. The 8% recurrence rate, while seemingly modest, carries meaningful clinical implications given the vulnerability of this age group, and may partly reflect an underlying pathological lead point or incomplete initial reduction that went undetected [39, 44]. The 2% perforation rate, though uncommon, underscores the need for careful pre-procedural patient selection, as perforations disproportionately occur in infants with longer symptom durations, significant bowel edema, or signs approaching peritonitis [42].

### Comparison with previous meta-analyses

Our findings are consistent with prior meta-analyses examining enema reduction for pediatric intussusception. Gray et al. (2014), documented a recurrence rate of 8.5% following ultrasound-guided non-contrast enema, which closely parallels our observed rate of 8% [12].

Liu et al. (2024) included 49 randomized and retrospective studies comprising over 9,000 patients [14]. In comparison to air reduction performed under fluoroscopic guidance, UGHR was linked to a shorter time to reduction, greater success rates, reduced hospital stay, and lower overall complication and perforation rates, with statistically significant differences observed across outcomes.

Earlier large-scale data by Sadigh et al. (2015), analyzing 102 studies (32,451 children), reported pooled success rates of 69.6% for liquid enema and 82.7% for air enema across all ages [49]. Our pooled success rate of 80% exceeds their liquid enema estimate. However, their analysis spanned nearly five decades of heterogeneous practice, including older, non–ultrasound-guided techniques. By focusing on modern, real-time UGHR in infants, our findings suggest that contemporary technique and imaging guidance substantially narrow the historical gap between liquid and pneumatic approaches.

More recently, A study by Qafesha et al. (2025) analyzed 29 studies comprising 9,281 patients and reported that pneumatic reduction demonstrated higher overall success rates and shorter reduction times compared with liquid enema [8]. However, no significant differences were observed in perforation, recurrence, hospital stay, or procedural parameters. Subgroup analyses showed higher pneumatic success in earlier studies (pre-2011), whereas more recent data demonstrated similar success rates between modalities. Outcomes were also comparable in centers with pediatric radiologists, suggesting that operator expertise may mitigate differences between techniques.

Our findings complement this body of evidence by focusing specifically on UGHR in infants, confirming its efficacy and acceptable safety within this distinct population.

### Interpretation and clinical implications

These findings carry important implications for clinical decision-making. An 80% success rate suggests that UGHR should be considered the first-line approach for hemodynamically stable infants younger than 12 months presenting with intussusception in centers with appropriate radiological and pediatric surgical expertise. Infants represent the population most vulnerable to cumulative radiation exposure; therefore, avoiding ionizing radiation alone strongly supports prioritizing ultrasound-guided techniques whenever feasible.

The 8% recurrence rate, while generally manageable through repeat enema reduction in most cases, reinforces the importance of structured post-reduction surveillance protocols. Clinicians should counsel caregivers on warning signs of recurrence and establish clear pathways for urgent re-evaluation. The 2% perforation rate, though low in absolute terms, highlights the critical importance of strict patient selection; infants with prolonged symptom duration exceeding 48–72 h, significant abdominal distension, or clinical signs of peritonitis should be referred directly for surgical evaluation rather than subjected to enema reduction attempts.

### Strengths and limitations

This study has several important strengths. To our knowledge, it is the first meta-analysis dedicated exclusively to infants under 12 months undergoing UGHR, generating age- and modality-specific estimates that previous reviews, often combining age groups or multiple techniques, were unable to provide. Additionally, the inclusion of studies from a broad range of geographic regions, including Asia, Africa, the Middle East, and Australia, improves the external validity of our findings across different healthcare contexts. Another strength is the use of subgroup analyses by sedation status, study design, and country income level, which provided further insight into potential sources of outcome variation. Finally, the application of a random-effects model appropriately addresses the clinical and methodological heterogeneity inherent in this body of literature.

Nevertheless, important limitations deserve acknowledgment. First, the majority of included studies were retrospective in design, and only a single RCT was identified, limiting the certainty of evidence, which the GRADE framework rated as low for all three outcomes. Second, the high statistical heterogeneity indicates that pooled estimates should be interpreted with appropriate caution, as they integrate studies differing in operator expertise, institutional protocols, patient selection criteria, and geographic context. Third, funnel plot asymmetry for success and recurrence rates raises the possibility of publication bias, with smaller studies reporting less favorable outcomes potentially underrepresented in the literature. Finally, confounding by symptom duration, intussusception subtype, and the use of sedation across studies could not be fully adjusted for in this analysis.

### Conclusion and recommendations

This meta-analysis demonstrates that UGHR achieves a clinically meaningful success rate of 80% in infants younger than 12 months, while maintaining an acceptably low perforation rate of 2% and a recurrence rate of 8%. Overall, hydrostatic reduction outcomes were largely unaffected by sedation status or study design, while perforation risk appeared to vary significantly by country income level. These findings support UGHR as an effective and safe nonoperative treatment option in appropriately selected infants; however, direct comparisons with alternative reduction techniques in this age group remain limited.

We recommend that institutions managing pediatric intussusception prioritize the development of standardized ultrasound-guided hydrostatic reduction protocols, particularly in settings where fluoroscopic guidance is unavailable or undesirable. Post-reduction observation and structured caregiver education regarding recurrence warning signs should be incorporated into routine discharge planning.

Given the persistent heterogeneity and low certainty of the current evidence base, well-designed multicenter prospective studies and randomized controlled trials comparing ultrasound-guided hydrostatic reduction with alternative techniques in infants younger than 12 months are urgently needed. Such studies should prospectively capture operator experience, sedation use, symptom duration, hydrostatic pressure parameters, and follow-up outcomes in a standardized fashion to better define predictors of success, recurrence, and perforation in this uniquely vulnerable population.

## Supplementary Information

Below is the link to the electronic supplementary material.

Supplementary Material 1

## Data Availability

Yes. This study analyzed previously published data through a systematic review and meta-analysis; no primary data were generated.
