# Supplementary material for: Ultrasound-guided hydrostatic enema reduction for intussusception in children younger than 12 months: a systematic review and meta-analysis
Source: Pediatr Surg Int. 2026 Jul 31;42(1):324. doi: 10.1007/s00383-026-06546-9 (PMC13427986; doi:10.1007/s00383-026-06546-9)

| **Database** | **Search strategy** |
| --- | --- |
| PubMed | ("ultrasound guided"[All Fields] OR "ultrasonography"[All Fields] OR "sonography"[All Fields]) AND ("hydrostatic enema"[All Fields] OR "saline enema"[All Fields] OR "fluid enema"[All Fields]) AND ("intussusception"[All Fields]) AND ("infant*"[All Fields] OR ("paediatrics"[All Fields] OR "pediatrics"[MeSH Terms] OR "pediatrics"[All Fields] OR "paediatric"[All Fields] OR "pediatric"[All Fields]) OR ("paediatrics"[All Fields] OR "pediatrics"[MeSH Terms] OR "pediatrics"[All Fields] OR "paediatric"[All Fields] OR "pediatric"[All Fields])) |
| Cochrane central | (("ultrasound guided" OR "ultrasonography" OR "sonography") AND ("hydrostatic enema" OR "saline enema" OR "fluid enema") AND ("intussusception") AND (infant* OR pediatric OR paediatric)) |
| Web of science | TS= (("ultrasound guided" OR "ultrasonography" OR "sonography") AND ("hydrostatic enema" OR "saline enema" OR "fluid enema") AND ("intussusception") AND (infant* OR pediatric OR paediatric)) |
| Scopus | TITLE-ABS-KEY (("ultrasound guided" OR "ultrasonography" OR "sonography") AND ("hydrostatic enema" OR "saline enema" OR "fluid enema") AND ("intussusception") AND (infant* OR pediatric OR paediatric)) |
| Embase | ((("ultrasound guided" OR "ultrasonography" OR "sonography") AND ("hydrostatic enema" OR "saline enema" OR "fluid enema") AND ("intussusception") AND (infant* OR pediatric OR paediatric)))/br |

**Table S1.** Search strategy across databases of effects of Ultrasound guided hydrostatic enema reduction on infants less than 12 months with intussusception.

**Table S2.** Quality assessment of the observational cohort and case-control studies.

| **Study ID** | **D1** | **D2** | **D3** | **D4** | **Comparability** | **D5** | **D6** | **D7** | **Quality** |
| --- | --- | --- | --- | --- | --- | --- | --- | --- | --- |
| **Qiang et al. 2026** | ★ | ★ |  | ★ |  | ★ | ★ | ★ | **Moderate** |
| **Soundharya et al. 2025** | ★ | ★ |  | ★ |  | ★ | ★ | ★ | **Moderate** |
| **Tang et al. 2024** | ★ | ★ | ★ | ★ |  | ★ | ★ | ★ | **High** |
| **Binu et al. 2023** | ★ | ★ |  | ★ |  | ★ | ★ | ★ | **Moderate** |
| **Ali et al. 2022** |  | ★ |  | ★ |  |  | ★ | ★ | **Low** |
| **Zhang et al. 2022** | ★ | ★ |  | ★ | ★ | ★ | ★ | ★ | **High** |
| **Yu et al. 2021** | ★ | ★ | ★ | ★ | ★ |  | ★ | ★ | **High** |
| **Chukwubuike et al. 2020** |  | ★ |  | ★ |  |  |  | ★ | **Moderate** |
| **Sarma et al. 2020** | ★ | ★ | ★ | ★ |  |  | ★ | ★ | **Moderate** |
| **Sacks et al. 2020** | ★ | ★ | ★ | ★ |  | ★ | ★ | ★ | **High** |
| **Shen et al. 2018** | ★ | ★ |  | ★ |  |  | ★ | ★ | **Moderate** |
| **Talabi et al. 2018** | ★ | ★ |  | ★ |  |  | ★ | ★ | **Moderate** |
| **Eraki et al. 2017** | ★ | ★ |  | ★ |  |  | ★ | ★ | **Moderate** |
| **Khorana et al. 2015** | ★ | ★ | ★ | ★ | ★ | ★ | ★ | ★ | **High** |
| **He et al. 2014** | ★ | ★ | ★ | ★ |  | ★ | ★ | ★ | **High** |
| **Atalabi et al. 2013** | ★ | ★ |  | ★ |  | ★ | ★ | ★ | **Moderate** |
| **Renzo et al. 2012** | ★ | ★ | ★ | ★ | ★ | ★ | ★ | ★ | **High** |
| **Mensah et al. 2011** | ★ | ★ |  | ★ |  |  | ★ | ★ | **Moderate** |
| **Choi et al. 1994** | ★ | ★ |  | ★ |  |  |  | ★ | **Low** |

**D1:** Is the case definition adequate representative of the exposed cohort?
**D2:** Representative of the cases/selection of the non exposed cohort?
**D3:** Selection of the controls and ascertainment of exposure
**D4:** Definition of controls/ Demonstration that outcome of interest aren’t present at the start of study
**D5:** Ascertainment of exposure/ Assessment of outcome
**D6:** Same method of ascertainment for cases and controls/ Was follow-up long enough for outcomes to occur
**D7:** Is the follow-up duration adequate?

**Table S3.** NIH assessment tool of case series studies.

| **ID** | **NIH Quality Assessment Tool for Observational Case series Studies** | | | | | | | | | |
| --- | --- | --- | --- | --- | --- | --- | --- | --- | --- | --- |
|  | **D1** | **D2** | **D3** | **D4** | **D5** | **D6** | **D7** | **D8** | **D9** | **Total scores** |
| **Atalabi et al. 2007** | Yes | Yes | Yes | No | Yes | NR | Yes | Yes | Yes | **Good** |

**D1:**Was the study question or objective clearly stated?
**D2:** Was the study population clearly and fully described, including a case definition?
**D3:** Were the cases consecutive?
**D4:** Were the subjects comparable?
**D5:** Was the intervention clearly described?
**D6:** Were the outcome measures clearly defined, valid, reliable, and implemented consistently across all study participants?
**D7:** Is the follow-up duration adequate?
**D8:** Were the statistical methods well-described?
**D9:** Were the results well-described?

**Table S4)** Quality assessment of the observational cross-sectional study.

| **Study ID** | **D1** | **D2** | **D3** | **D4** | **Comparability** | **D5** | **D6** | **D7** | **D8** | **Score** |
| --- | --- | --- | --- | --- | --- | --- | --- | --- | --- | --- |
| **Yadav et al. 2025** | ★ | ★ |  | ★ |  | ★ |  |  | ★ | **Moderate** |

**D1:** Clearness of Aim. **D2:** Representativeness of the cases. **D3:** Sample size. **D4:** missing rate. **D5:** Exposure Assessment. **D6:** Confounding Factors. **D7:** Outcome Assessment. **D8:** Statistical Tests

**Table S5)** Quality assessment of Randomized controlled trial of Ultrasound Guided Hydrostatic enema reduction in pediatric intussusception.

| **Study ID** | **The Cochrane Collaboration’s tool for assessing risk of bias** | | | | | | |
| --- | --- | --- | --- | --- | --- | --- | --- |
|  | **D1** | **D2** | **D3** | **D4** | **D5** | **D6** | **D7** |
| Chukwu et al. 2022 | Low risk | Unclear | Low risk | High risk | Low risk | Low risk | High risk |

**D1:** Random sequence generation (Selection bias)
**D2:** Allocation concealment (Selection bias)
**D3:** Blinding of participants and personnel (Performance bias)
**D4:** Blinding of outcome assessment (Detection bias)
**D5:** Incomplete outcome data (Attrition bias)
**D6:** Selective reporting (Reporting bias)
**D7:** Other Bias

**Table S6.** Grade assessment of Ultrasound-guided hydrostatic enema reduction for pediatric intussusception.

| **Certainty assessment** | | | | | | | |
| --- | --- | --- | --- | --- | --- | --- | --- |
| **№ of studies** | | **Study design** | **Risk of bias** | **Inconsistency** | **Indirectness** | **Imprecision** | **Certainty** |
| **Success rate** | | | | | | | |
| 18 | 18 Retrospective studies and one RCT | | Some concern | Serious due to high heterogeneity | Not serious | Not serious | Low |
| **Recurrence rate** | | | | | | | |
| 14 | 13 Retrospective studies and one RCT | | Some concern | Serious due to high heterogeneity | Not serious | Not serious | Low |
| **Perforation rate** | | | | | | | |
| 7 | Seven Retrospective studies and one RCT | | Some concern | Serious due to heterogeneity | Not serious | Not serious | Low |

**RCT:** Randomized Controlled Trial.

**Figure S1.** Forest plot of the subgroup analysis of success rate by sedation status.


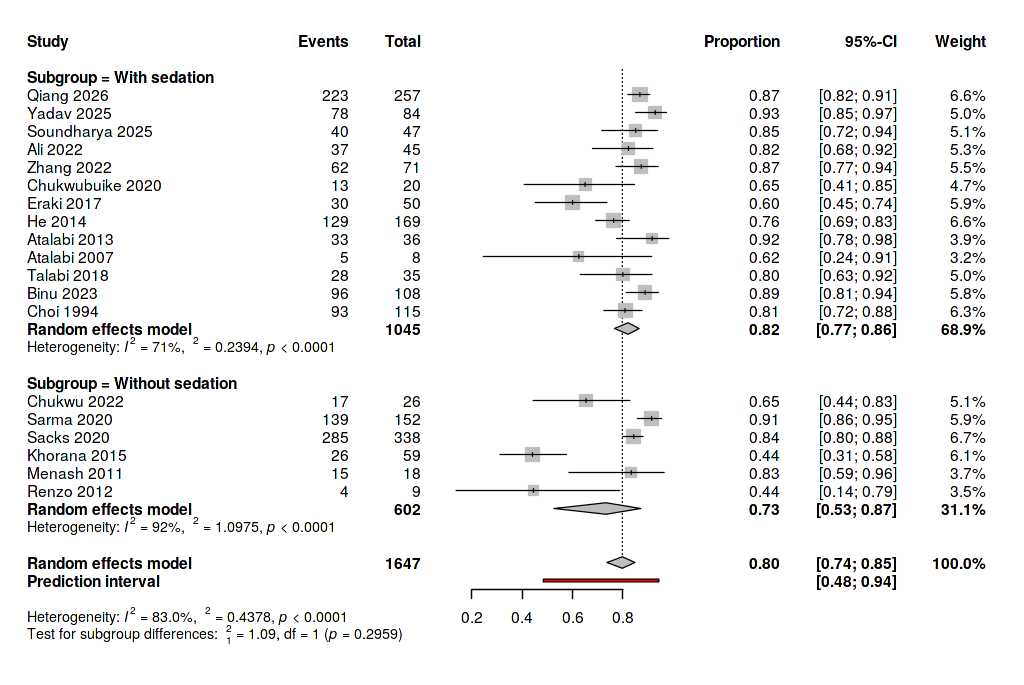


**Figure S2.** Forest plot of the subgroup analysis of success rate by study design.


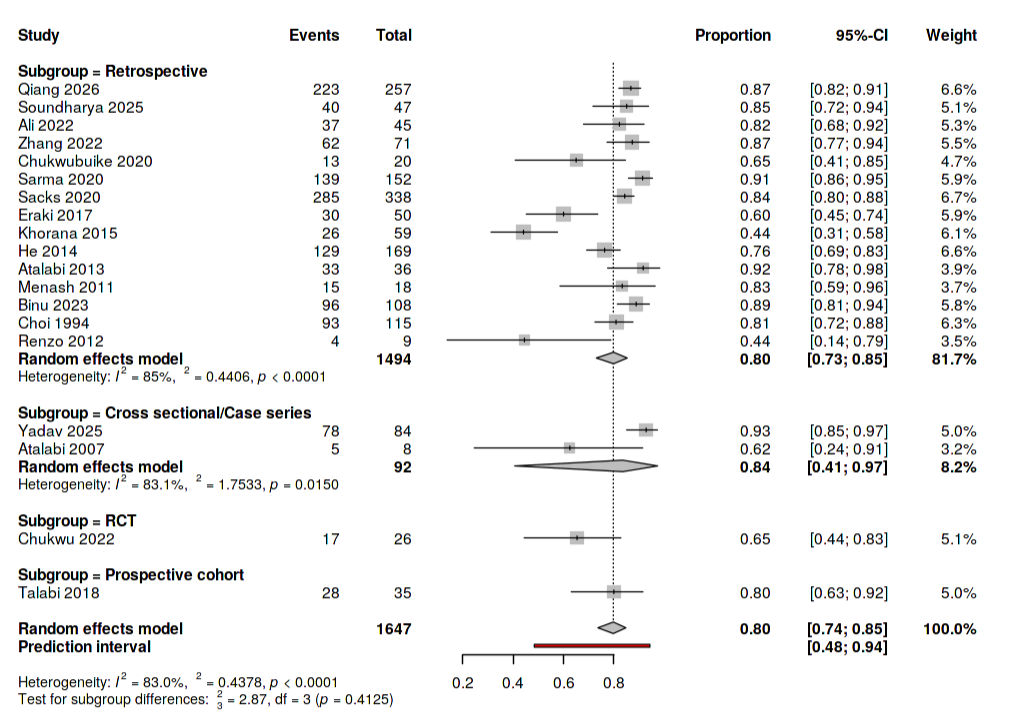


**Figure S3.** Forest plot of the subgroup analysis of success rate by country income level.


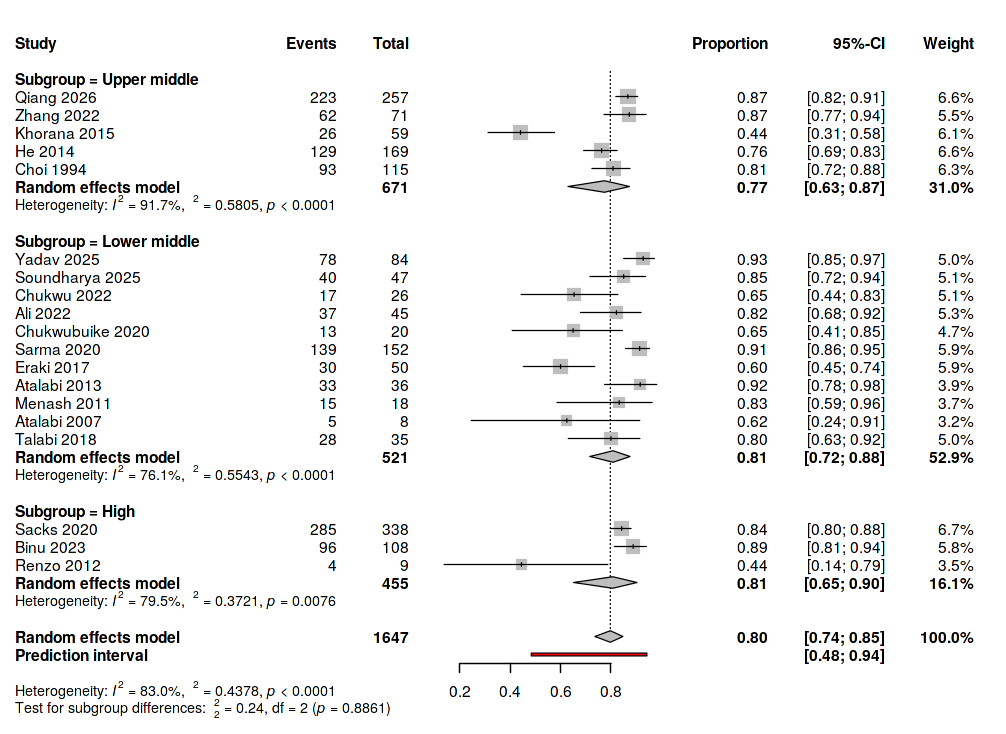


**Figure S4.** Forest plot of the subgroup analysis of recurrence rate by sedation status.

**
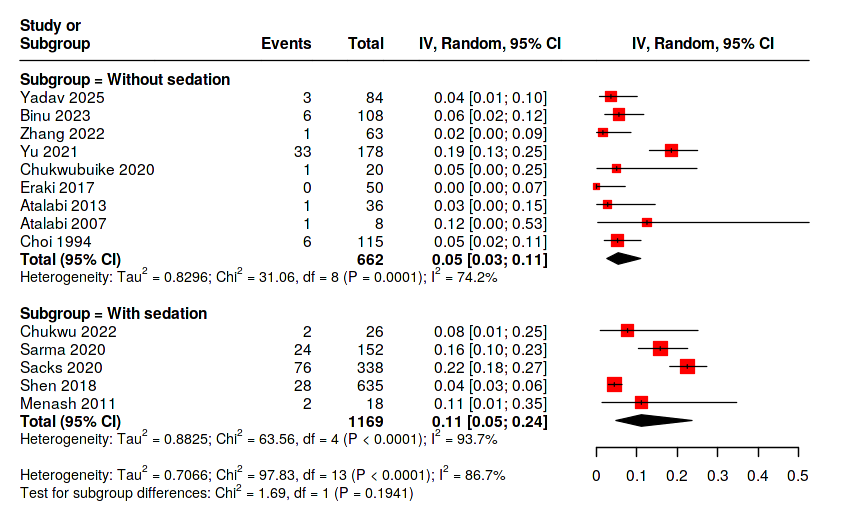
**

**Figure S5.** Forest plot of subgroup analysis of recurrence rates by study design.


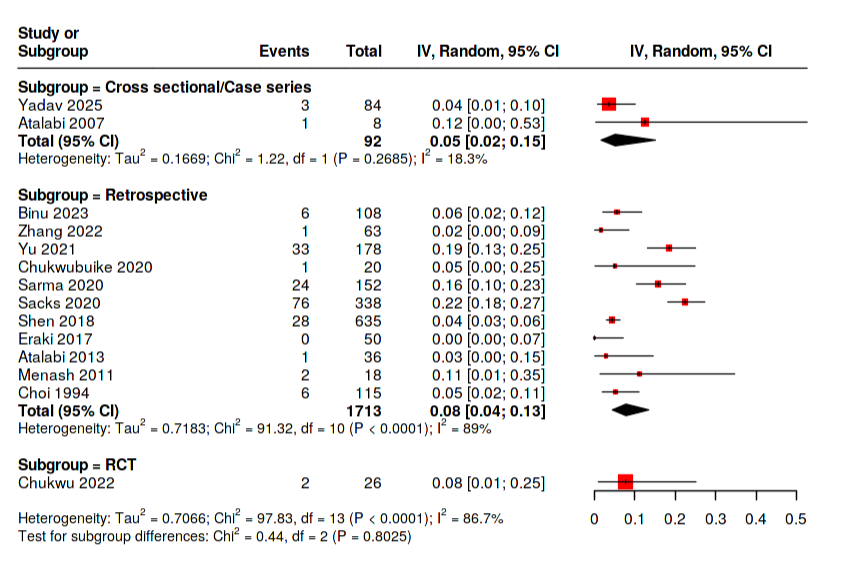


**Figure S6.** Forest plot of the subgroup analysis of recurrence rate by country income level.


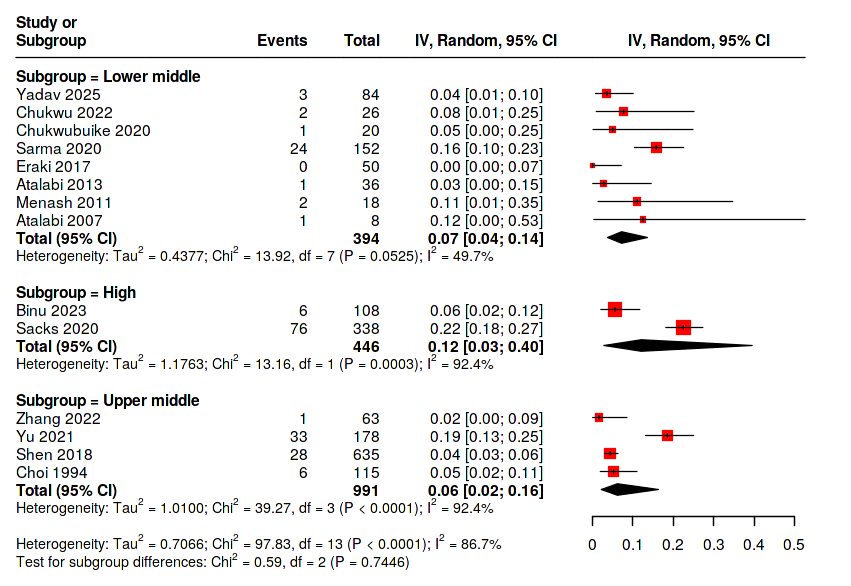


**Figure S7.** Forest plot of the subgroup analysis of perforation rate by sedation status.


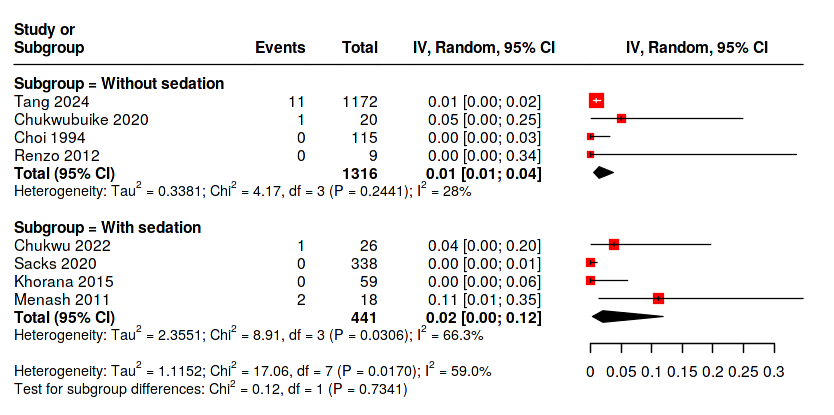


**Figure S8.** Forest plot of the subgroup analysis of perforation rate by study design.


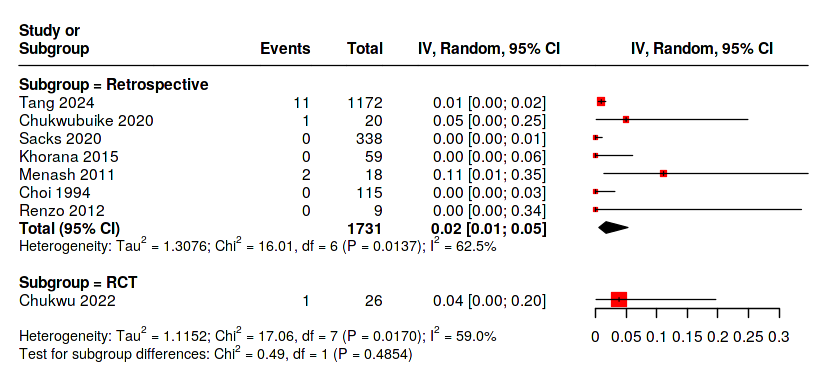


**Figure S9.** Forest plot of the subgroup analysis of perforation rate by country income level.


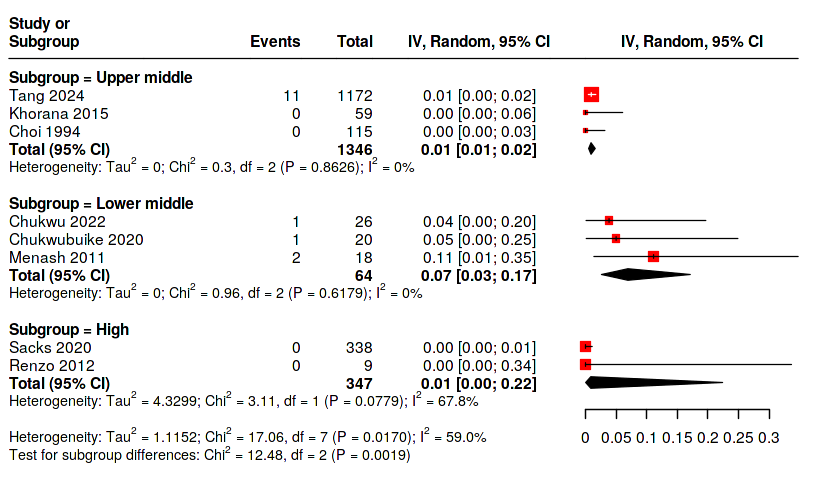


**Figure S10.** Funnel plot for the pooled success rate.

**
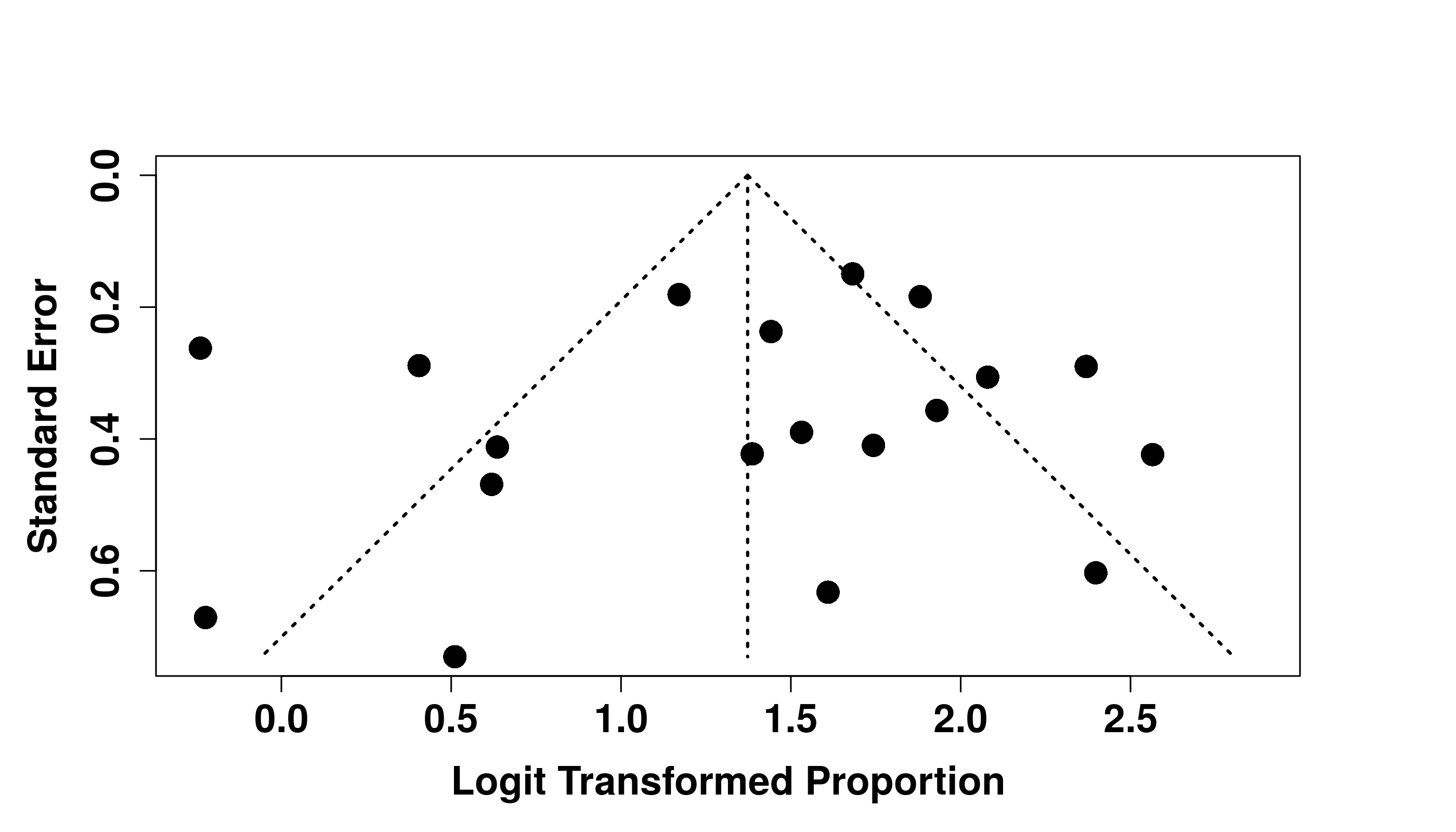
**

**Figure S11.** Funnel plot for the pooled recurrence rate.


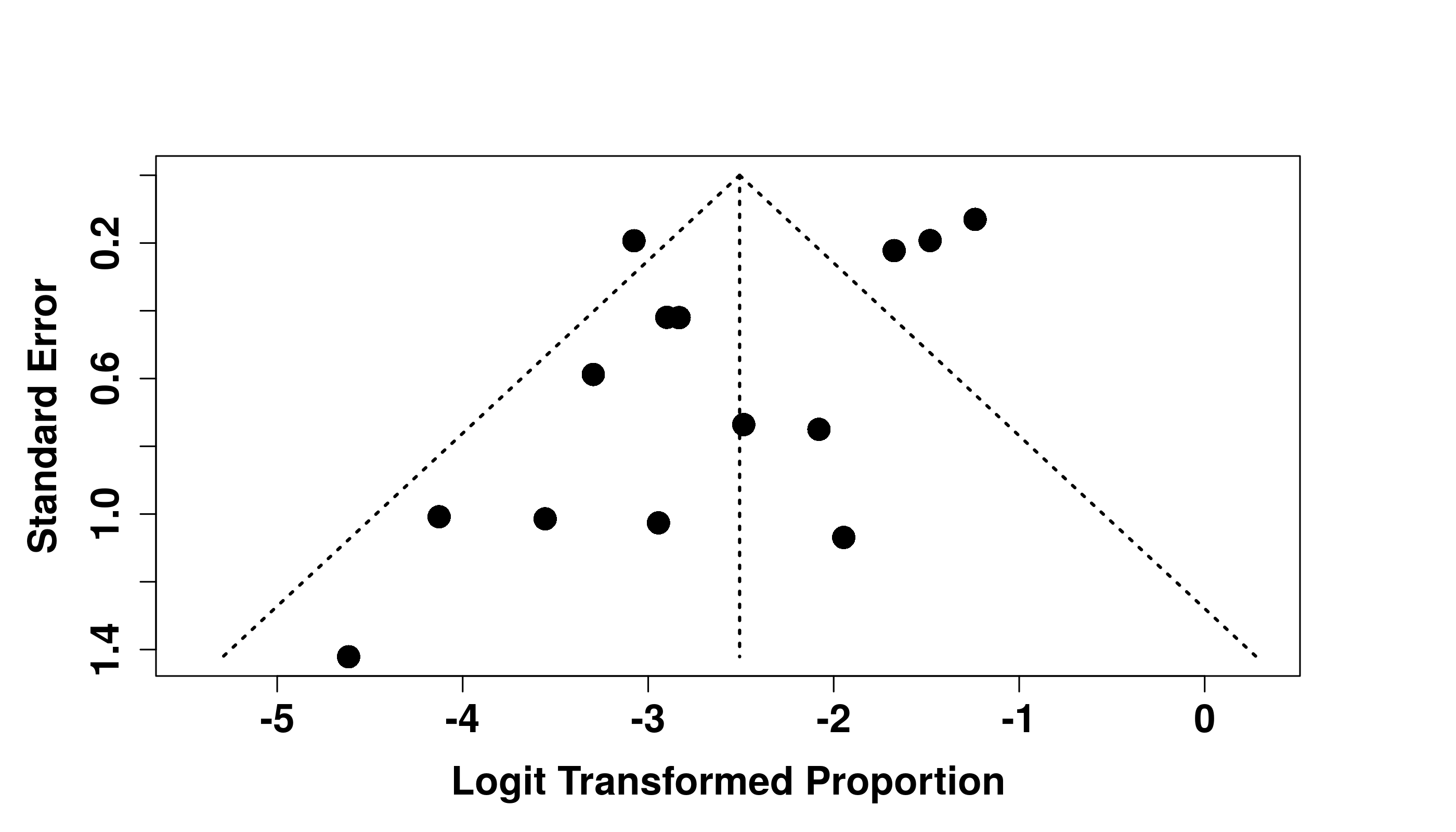


**Figure S12.** Funnel plot for the pooled perforation rate.


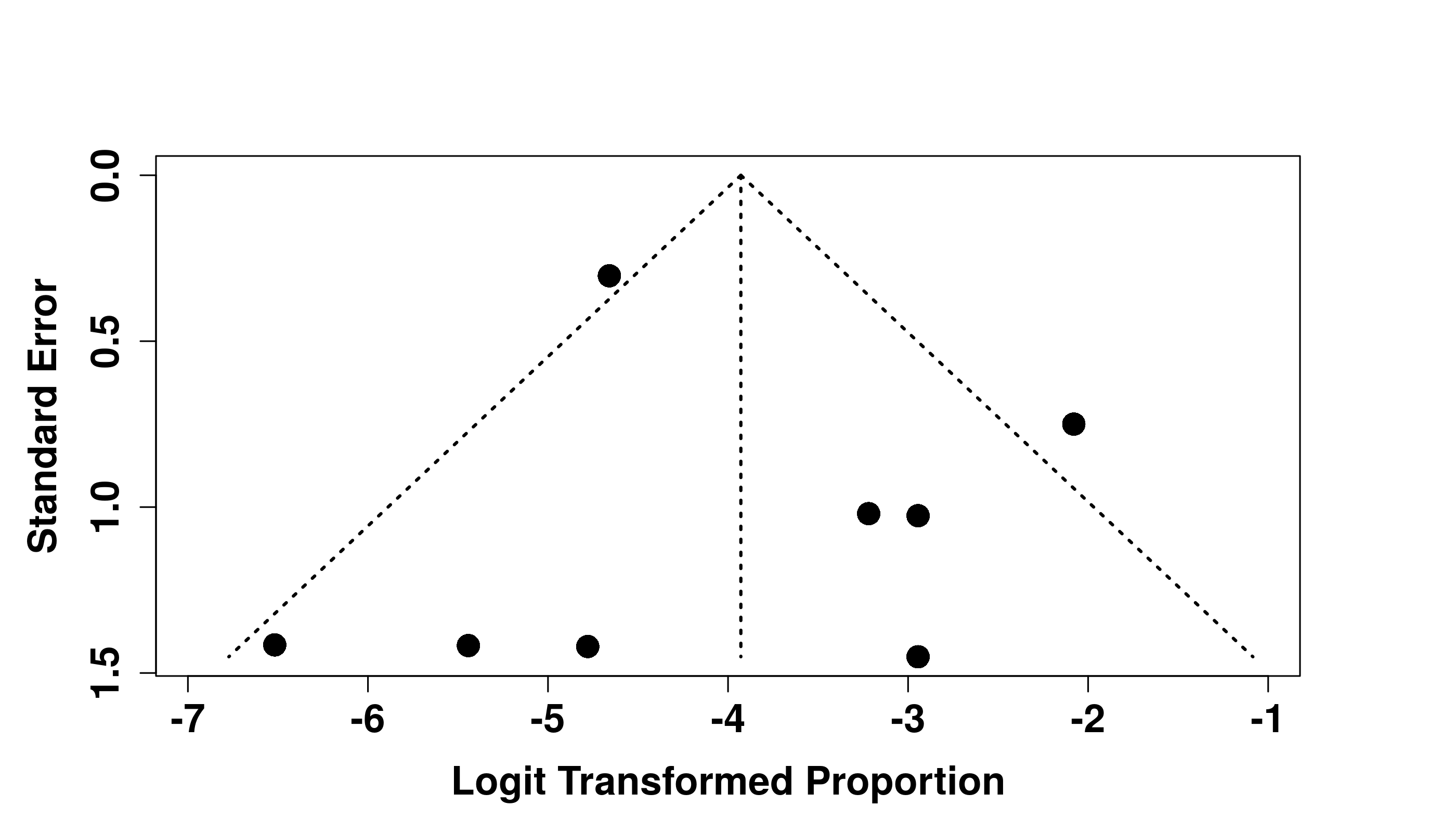

Supplement: Supplementary file 1 — Supplementary Material 1 [file 383_2026_6546_MOESM1_ESM.docx]
